# Transactivation of human endogenous retroviruses by tumor viruses and their functions in virus-associated malignancies

**DOI:** 10.1038/s41389-018-0114-y

**Published:** 2019-01-14

**Authors:** Jungang Chen, Maryam Foroozesh, Zhiqiang Qin

**Affiliations:** 1 Department of Pathology, Winthrop P. Rockefeller Cancer Institute, University of Arkansas for Medical Sciences, 4301 W Markham St, Little Rock, AR 72205 USA; 2 Department of Chemistry, Xavier University of Louisiana, 1 Drexel Drive, New Orleans, LA 70125 USA; 3 Department of Pediatrics, East Hospital, Tongji University School of Medicine, 200120 Shanghai, China; 4 Research Center for Translational Medicine and Key Laboratory of Arrhythmias, East Hospital, Tongji University School of Medicine, 200120 Shanghai, China

## Abstract

Human endogenous retroviruses (HERVs), viral-associated sequences, are normal components of the human genome and account for 8–9% of our genome. These original provirus sequences can be transactivated to produce functional products. Several reactivated HERVs have been implicated in cancers and autoimmune diseases. An emerging body of literature supports a potential role of reactivated HERVs in viral diseases, in particular viral-associated neoplasms. Demystifying studies on the mechanism(s) of HERV reactivation could provide a new framework for the development of treatment and prevention strategies targeting virus-associated tumors. Although available data suggest that co-infection by other viruses, such as Kaposi’s Sarcoma-associated herpesvirus (KSHV) and Epstein–Barr virus (EBV), may be a crucial driving force to transactivate HERV boom, the mechanisms of action of viral infection-induced HERV transactivation and the contributions of HERVs to viral oncogenesis warrant further studies. Here, we review viral co-infection contributes to HERVs transactivation with focus on human viral infection associated oncogenesis and diseases, including the abilities of viral regulators involved in HERV reactivation, and physiological effects of viral infection response on HERV reactivation.

## Background

Human endogenous retroviruses (HERVs) are a subgroup of retroviruses integrating their sequences into host genome after exogenous retrovirus infection millions of years ago, which account for about 8–9% of human genome^1,2^. Due to the accumulation of mutation, most HERVs are commonly inactive and unable to replicate. However, some HERVs still have open reading frames and keep a potential for protein expression^3,4^. A growing number of findings suggest that viral products of HERVs may have a role in species evolution, as well as various diseases^3,5–7^.

**Fig. 1:**
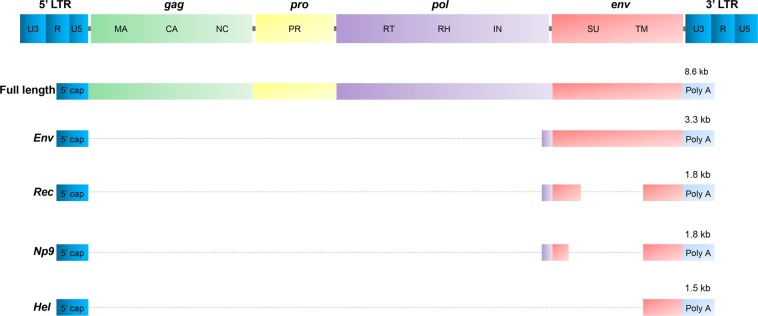
Diagrams of HERV-K proviruses and their transcripts. A compete sequence of HERVs are composed of *gag, pol*, *pro*, and *env* regions sandwiched between two long terminal repeats (LTRs). *Gag* encodes the structural components of matrix (MA), capsid (CA), and nucleocapsid (NC). The products of *pol* gene are reverse transcriptase (RT), integrase (IN), and RNase H (RH). The *pro* mainly encodes the enzyme protease (PR), while *env* encodes Env surface (SU) and transmembrane (TM) subunits. LTRs are composed of U5 region, U3 region and repeat sequences (R). The HERV-K (HML-2) usually expresses a full-length transcript (8.6-kb) and encodes the gag, pro, and pol polyproteins. *Env* gene transcripts two singly spliced products, a 3.3-kb product to encode Env polyprotein, and a 1.5-kb product of unknown function known as the *hel* transcript, and a doubly spliced product (1.8-kb) to encode either the Rec or Np9 accessory proteins depending on the presence or absence of a 292-bp deletion at the *pol*/*env* boundary

Retroviruses are double-stranded positive-sense RNA viruses encoding and carrying reverse transcriptase (RT) to reversely transcribe RNAs to DNAs. These viral DNAs are then integrated into the host DNA mediated by its integrase enzyme (IN), thus creating a provirus, which can translate and transcribe viral products^8,9^. Similar to integrated retrovirus, a complete sequence of HERVs are mainly composed of *gag*, *pro*, *pol*, and *env* regions sandwiched between two long terminal repeats (LTRs) (Fig. 1). LTRs contain main promoters, enhancers, and transactivation regions for HERV transcription, thus regulating activation and expression of HERV genes^10^. The *gag* and *pol* usually encode polyproteins, which are then processed into individual proteins. The products of *gag* are structural proteins and *pol* codes for the RT, IN, and RNAse H. Notably, unlike HIV genome, the *pro* gene of HERVs is separated from the *pol* reading frame. The product of *env* gene is a glycosylated protein and is cleaved into two viral envelope proteins, a surface unit (SU) and a transmembrane unit (TM)^3,11,12^.

Currently, HERVs are classified into 22 independently acquired families based on the first-letter amino-acid core of the tRNA of the primary binding site used by HERV to start reverse transcription^13^. Of these, some HERV families, such as HERV-K, were identified to be relevant with the development of human cancers, such as breast cancer^14^, lung cancer^15^, prostate cancer^16^, hepatocellular carcinoma (HCC)^17^, melanomas^18^, germ cell tumor^19^, leukemia^20^ and, lymphoma^21^. HERV-K is the most recent HERV family acquired by humans at around three million years ago^22^. In contrast to all other HERVs, some HERV-Ks are to date the only known human endogenous proviruses that have retained open reading frames for all viral proteins, such as HERV-K (HML-2)^8,23^. HERV-Ks are formed by 11 subgroups (HML-1-HML-11), with the most-studied one in cancers being HERV-K (HML-2)^8^. HERV-K (HML-2) has two major types of proviruses (type I and II), for which the nomenclature is based on the presence (type I) or absence (type II) of a 292-bp deletion at the *pol*/*env* boundary encoding two variant proteins, Np9 and Rec, respectively^11,24^. The type II provirus produces the regulatory protein Rec by a singly spliced transcript, while the type I provirus produces Np9 through a doubly spliced transcript in the *pol*/*env* boundary region. HML-2 also expresses a 1.5-kb transcript with unknown function referred to as the *hel* transcript^11,12^ (Fig. 1). Furthermore, both Rec and Np9 have been reported as oncogenic proteins and are present in a variety of tumors and transformed cells.

Although the precise role of HERVs in development of tumors has not been fully elucidated, there are increasing data suggesting that HERVs are closely related to human malignancies. Many studies have identified high levels of the expressed products of HERVs in cells, tissues, and blood of patients with cancers^14–22^. The transactivation of HERVs may affect carcinogenesis process through directly expressing viral mRNA, functional proteins, and/or viral particles, or indirectly activating tumor-associated genes. Viral products of many HERVs, such as the K, H, R, and T families, have been detected in cells, blood and tissues of patients with lung cancer or breast cancers. Levels of HERVs transactivation have been shown to be much higher in these patients than those in healthy volunteers^25–27^. The positive correlation of HERVs transactivation with cancer is strongly supported by the observation that some specific antibodies or shRNAs against HERV-K possess inhibitory effect on the growth of cancer cells in vitro and in vivo^28,29^. Thus, HERVs could be considered as suitable prognostic markers for a variety of malignant diseases, such as lung cancers and HCC^17,25^. Additional studies have found that Np9 and Rec proteins of HERV-K physically and functionally interact with the promyelocytic leukemia zinc finger (PLZF) tumor suppressor to regulate cancer cell proliferation and survival through altering the expression of the c-*Myc* proto-oncogene^30,31^.

## Viral infection and HERVs transactivation

Although the detailed mechanisms of HERVs transactivation remain largely unclear, a variety of inducers have been reported, including some external and internal signals. Of these, viral infection plays important roles in the regulation of HERVs transactivation (Table 1). Many recent studies have shown that infection with exogenous viruses, such as HIV-1, HBV, HTLV-1, Influenza A virus, and herpesviruses, can induce significant HERVs transactivation, which in turn, co-contributes to the development of viral diseases, including virus-associated tumors^32–37^. For instance, many HERVs are activated in HIV-1-infected patients, and the levels of HERV products are decreased in patients with anti-HIV treatment^38^. Studies on mechanisms of HERV-K transactivation show that HIV-1 Tat protein can induce HERV-K expression through regulating the NF-κB and NF-AT pathways^39,40^. Also, Influenza A/WAN/33 virus infection can induce transcriptional de-repression of the ERVWE1 of HERV-W by increasing transcription of GCM1 and reducing H3K9me3^41^. Another example is that of Herpes simplex virus 1 (HSV-1) infections, which can activate both HERV-W and HERV-K through two different pathways, in which viral IE1 enhances the activity of Oct-1 to stimulate HERV-W^42^, while ICP0 upregulates the activity of AP-1 to activate HERV-K^43^. In fact, ~20% of human cancers have been found to be related to viral infections, but the mechanism of viral oncogenesis is largely unclear. However, recent data about HERVs transactivation induced by tumor viruses and their function in malignant diseases indicate that HERVs transactivation may act as potential regulators or co-contributors to viral oncogenesis. Here, we present a summary of recent findings regarding the relationship of different tumor viruses with HERVs transactivation.

**Table 1:** Viral infections induced HERVs transactivation

Viruses	HERV family	Possible mechanisms	Ref.
HSV-1	W, K	IE1 stimulates LTR of HERV-W trough enhancing the activity of Oct-1; ICP0 increases transcription LTR of HERV-K through AP-1 site.	^36, 42, 43^
VZV	Unknown	VZV can sustain the increase in the RT expression.	^89^
HCMV	T, W, F, K, L	HCMV-induced cytokines and growth factors may enhance HERV activation.	^68, 69^
EBV	W, K	LAM-2A and LMP-1 activate HERV-K in infected B lymphocytes; EBV infection activates HERV-K in resting B lymphocytes through binding CD21; HERV-W activation was regulated by EBV gp350 in PBMC.	^37, 57– 59, 90^
HHV-6	K	HHV-6A induces HERV-K18-encoded superantigen through IFN-α; HHV-6B induced superantigen HERV-K18, which may have consequences for the development of autoimmunity.	^91, 92^
KSHV	K	LANA induces env transcripts through enhancing ERK activity; vFLIP induces env transcripts through activating NF-κB activity.	^33^
HIV-1	K, E, W, T	HERV-K (HML-2) is activated by Tat through regulating NF-κB and NF-AT.	^2, 32, 40^
HTLV-1	K, E, W, H	Tax is able to activate HERV LTRs, mainly of HERV-W and -H.	^35, 74^
HBV	W	HBV X Protein induces overexpression of HERV-W env through NF-κB.	^34^
Influenza A virus	W	Influenza A virus infection can transactivate ERVWE1 by increasing the transcription of GCM1 and reducing the repressive histone mark H3K9me3.	^36, 41^

## Tumor virus infections and HERVs transactivation

### KSHV infection and HERVs transactivation

Kaposi’s sarcoma-associated herpesvirus (KSHV) is a double-strand DNA virus classified as a type 8 member of human herpesvirus family (HHV-8)^44^. Previous studies have confirmed that KSHV infection is capable of causing Multicentric Castleman’s disease (MCD) and several cancers, such as Kaposi’s sarcoma (KS), and primary effusion lymphoma (PEL)^45^. KSHV-induced KS is one of the most common acquired immuno-deficiency syndrome (AIDS)-associated tumors. Despite recent progress in the development of treatments for KSHV-associated malignancies, more effective therapies remain urgently needed.

KSHV infection has two alternative life cycle programs, latent and lytic phases, both of which can contribute to the development of KSHV-induced cancers^46^. Generally, latent infection is established and persists in host cells following KSHV de novo infection, with only a small population of cells undergoing spontaneous lytic replication in a temporally ordered manner. During latency, only a limited number of latent genes, such as *ORF71* (*v-FLIP*), *ORF72* (*v-Cyclin*), *ORF73* (*LANA*), *K12* (*Kaposin*), and viral miRNAs are constitutively expressed to be involved not only in the maintenance of viral genome stabilization, but also in the regulation of host microenvironment. Of the latent gene products, LANA and v-FLIP play critical roles in viral pathogenesis, especially KSHV-induced tumorigenicity^47^.

KSHV-induced tumors are found most frequently in HIV-1-infected or other immunosuppressed patients^48^. HERVs have also been associated with HIV-1-infected and autoimmune diseases^6,32^. Thus, these data hint the potential relevance of KSHV infection with HERVs expression. Interestingly, the hypothesis is supported by the observation that the high levels of HERV-K (HML-2) *env* transcripts has been found in peripheral blood mononuclear cell (PBMC) from KSHV-infected HIV + patients^33^. Although HIV-1 is one of the viral factors inducing HERVs transactivation and HIV-1 Tat promotes expression of HERVs transcripts through regulating NF-κB and NF-AT signals, the level of HERV *env* transcripts are much lower in PBMC from HIV + patients without KSHV co-infection, suggesting that KSHV is also an activator or co-factor of HERVs transactivation.

**Fig. 2:**
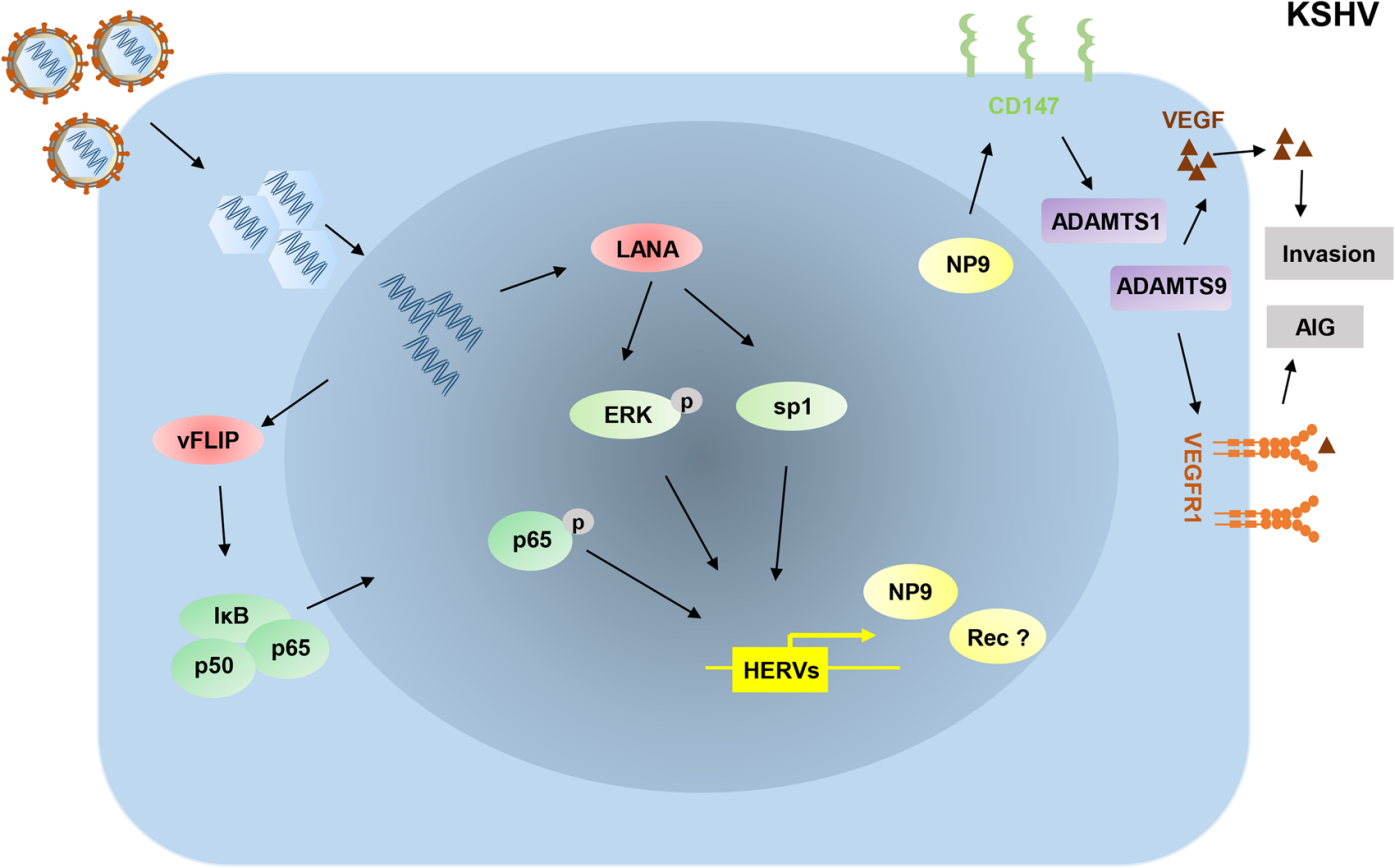
Schematic diagram of potential mechanisms for KSHV promoting HERV transactivation. During KSHV de novo infection, LANA induces *env* transcripts through enhancing ERK activity, and vFLIP induces *env* transcripts through activating NF-κB activity. Np9 expression mediated by KSHV can promote virus-induced anchorage-independent growth (AIG) and invasion through the CD147-ADAMTS1/ADAMTS9-VEGF/VEGFR1 axis. LANA: a latency-associated nuclear antigen; vFLIP: viral FADD-like interleukin-1-β-converting enzyme (FLICE)/caspase-8-inhibitory protein; ERK: extracellular-signal-regulated kinase; ADAMTS1: a disintegrin and metalloproteinase with thrombospondin motifs 1; ADAMTS9: a disintegrin and metalloproteinase with thrombospondin motifs 9; VEGF: vascular endothelial growth factor; VEGFR1: vascular endothelial growth factor receptor 1

Additional experimental data support that HERV-K (HML-2) transactivation is closely related to KSHV infection. The significantly higher levels of transcriptional products of HERV-K (HML-2) are found in KSHV + PEL tumor cells and KSHV de novo infected endothelial cells when compared to virus-negative control cells^33^. However, the levels of HERV-K associated transcripts are almost not changed in UV-inactivated KSHV-infected cells, implying HERV-K transactivation by KSHV infection may require the expression of KSHV latent transcripts. Mechanistic studies on KSHV-activated HERV-K (HML-2) show that two viral latent proteins, LANA and v-FLIP, regulate the transcription of HERV-K through both classical intracellular signaling pathways and cellular transcriptional factors (Fig. 2). LANA induces HERV-K *env* transcription through enhancing ERK signaling activity^33^. Furthermore, LANA may regulate HERV-K LTRs, which contain potential binding sites for viral and cellular transcriptional factors, through directly interacting with Sp1, a classical modulator of HERV-K LTR activities^33^. In fact, some other mechanisms, including DNA methylation, histone modification and the Rb (retinoblastoma) pathway, are also involved in the regulation of HERV-K transactivation^33^. Interestingly, LANA has been found to interact with or regulate Rb/E2F pathway and many epigenetic factors, such as EZH2, KDM3a, and DNMT3a^49,50^. Therefore, these additional mechanisms need to be further investigated. HERV-K *env* transcripts are also upregulated by another KSHV-encoded latent protein, v-FLIP, potentially through the activation of NF-κB pathway^33^.

HERV-K *env* transcripts encode two oncogenic proteins, Rec and Np9, both of which can promote cancer development. However, more prominent expression of Np9 than Rec has been found in KSHV-infected cells and AIDS-KS tumor tissues^33^. Moreover, Np9 is closely related to KSHV-induced invasion and anchorage-independent growth of primary endothelial cells through the regulation of the CD147-ADAMTS1/ADAMTS9-VEGE/VEGFR1 axis, enhancing viral pathogenesis in infected cells. Interestingly, silencing Np9 by RNAi in KSHV-infected TIVE-LTC cells dramatically reduced cell growth in vitro and suppressed the formation of KSHV-induced tumors in nude mice, suggesting that Np9 protein is an important co-factor for KSHV-induced tumorigenesis^33^. Therefore, the detailed function of HERVs transactivation in KSHV-related cancer progression, which may represent a promising direction for developing targeted therapy for KSHV-associated malignancies, needs to be further investigated.

### EBV infection and HERVs transactivation

Epstein–Barr virus (EBV), the type 4 member of the HHV family, is a ubiquitous virus. Studies show that up to 95% of all adults in the world have antibodies against this virus^51^. Previous studies confirmed that EBV infection has been linked to a number of malignant diseases, such as infectious mononucleosis, Burkitt’s lymphoma, Hodgkin’s lymphoma, naso-pharyngeal cancer, NK/T-cell lymphoma, post-transplant lymphoma, and multiple sclerosis^44^.

Similar to other herpesviruses, EBV infection has two alternative life cycle programs, latent and lytic phases^52^. While the lytic replication of EBV is pivotal to viral transmission and genome maintenance, the latency makes a more direct contribution to lymphoproliferative diseases^53^. EBV latent infection is established and persists in B cells and epithelial cells, however different latency programs are possible in these two types of cells. Based on which latent genes are expressed, latency of EBV can be divided into three distinct stages, Latency I, II, or III^54^. The latent gene products mainly include Epstein–Barr nuclear antigen 1 (EBNA1)/EBNA2/EBNA3A, EBNA3B, and EBNA3C, latent membrane protein 1 (LMP-1)/LMP-2A and LMP-2B, nuclear antigen leader protein, and virus-encoded small RNAs (EBERs), all of which are involved in the regulation of host gene expression and viral pathogenesis^53^.

**Fig. 3:**
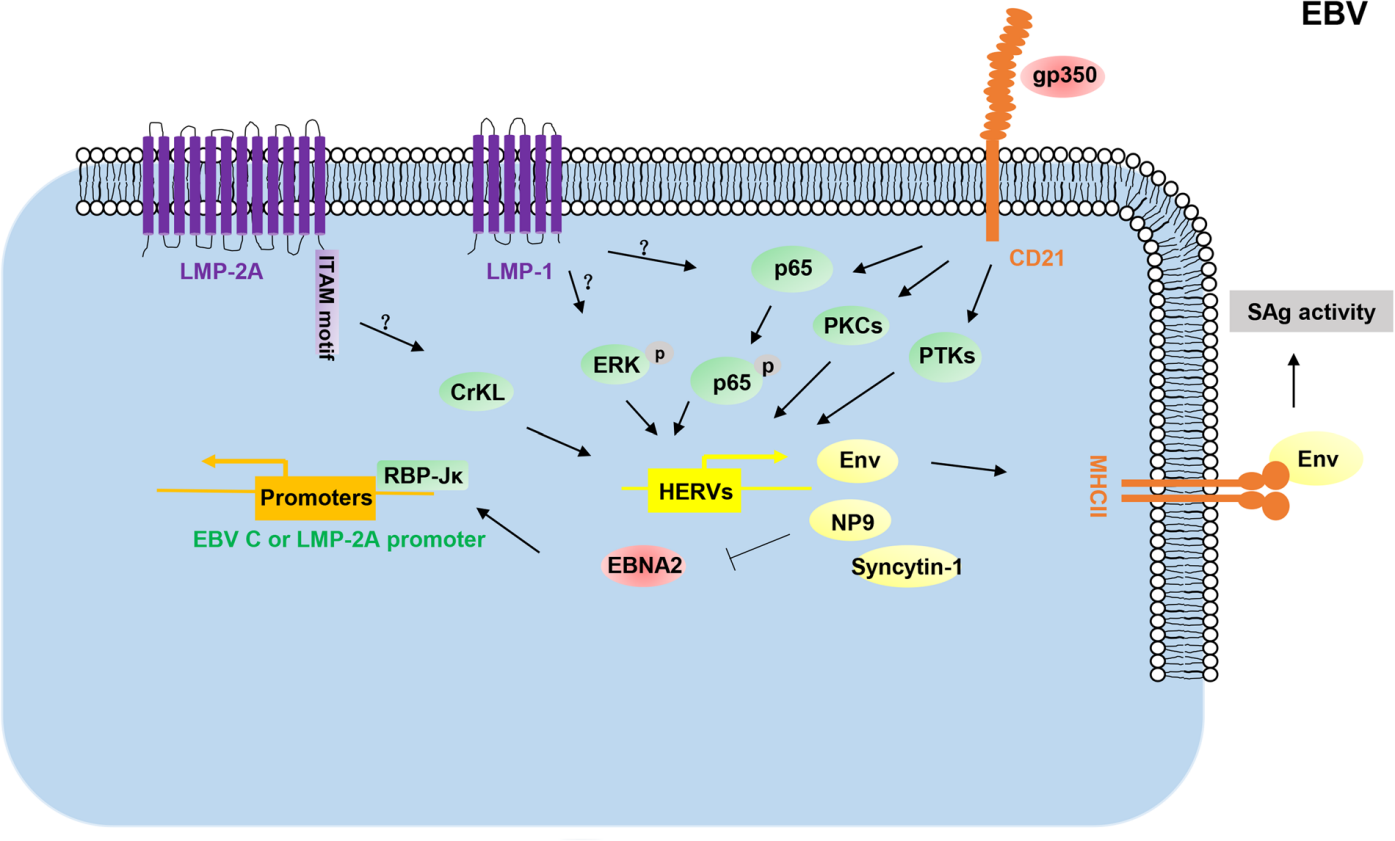
Schematic diagram of potential mechanisms for EBV promoting HERV transactivation. EBV infection can activate HERV expression through its gp350 protein interaction with its cellular receptor CD21 in the resting B-lymphocytes and PBMC. In infected B-lymphocytes, viral LAM-2A and LMP-1 activate the expression of HERV-K as superantigens (SAgs) to activate T-cell-mediated SAgs immune response. HERV-K Np9 binds to EBNA2 and negatively affects the EBNA2-mediated activation of the viral C- and LMP2A promoters. LAM-2A: Latent membrane proteins 2A; ITAM motif: an immunoreceptor tyrosine-based activation motif; LMP-2A: LMP-1 latent membrane proteins 1; EBNA2: viral nuclear antigen 2; gp350: glycoprotein 350

EBV infection usually induces superantigens (SAgs)-activated T-cell immune response^55^. T-cell activation mediated by SAgs plays important roles in viral maintenance and the development of virus-associated diseases^55,56^. Interestingly, Sutkowski et al. found that EBV infection transactivates the expression of HERV-K18 *env* gene that possesses SAg activity, which was further demonstrated by MHC class II dependent preferential activation of TCRVB13 T cells in response to murine B cells transfected with the HERV-K18 *env* gene^37^. Further studies revealed that EBV transactivates the HERV-K18 SAg through viral latent protein LMP-2A, LMP-1, and its cellular receptor, CD21^57,58^. While LMP-2A and LMP-1 each contribute to the induction of the SAg activity of HERV-K18 *env* gene in latently infected cells in vitro, EBV-encoded gp350 protein also triggers the expression of HERV-K18 *env* gene in resting B cells through binding to human CD21^58^. Data show that the immunoreceptor tyrosine-based activation motif (ITAM) of LMP-2A is important for HERV-K18 *env* transactivation through CrKL pathway^57^. The activation of ERK and NF-κB pathways may be important steps in LMP-1-mediated HERV-K activation, whereas gp350 activates HERV-K through protein kinase C, protein tyrosine kinase, and NF-κB pathways^58^ (Fig. 3). A recent study found that EBV-encoded gp350 also activates HERV-W/syncytin-1 in cells derived from blood and brain through the NF-κB pathway or some pro-inflammatory cytokines^59^, implying that HERV-W may be a potent contributor involved in the pathogenesis of multiple sclerosis.

Interestingly, HERV-K transactivation induced by EBV infection may in turn regulate viral gene expression. One study shows that HERV-K Np9 is strongly upregulated in EBV-transformed lymphocytes and is detected in many EBV + tumor cells^60^. These data show that Np9 protein is able to hijack EBNA2 to reduce the binding ability of EBNA2 to DNA-bound RBP-Jκ leading to the downregulation of the EBNA2-mediated activation of the viral C- and LMP-2A promoters^60^ (Fig. 3). Inhibitory effect of EBV-induced Np9 on viral gene expression may represent a protective mechanism, which controls excessive expression of viral products to promote proliferation of infected cells.

### HCMV infection and HERVs transactivation

Human cytomegalovirus (HCMV), a double-strand DNA virus, belongs to HHV family (also known as human herpesvirus-5, HHV-5)^61^. HCMV remains in latent phase within the body throughout life following primary infection, but it can be reactivated at any time^62^. HCMV infection is typically unnoticed in healthy people, but may cause life-threatening diseases in immunocompromised hosts, such as HIV-infected persons, organ transplant recipients, or newborn infants^63^. Increasing data show that HCMV may possess oncogenic or onco-modulatory functions in human cancers, because of its high prevalence in cancers of different origin, such as glioblastoma, medulloblastoma, neuroblastoma, colon, breast, and prostate cancers, and its ability to control the expression of host genes, especially the activation of cellular oncogenes and inhibition of tumor suppressor genes^64–67^.

Recent studies have revealed that in GliNS1 cells, HCMV infection regulates the transactivation of HERV-T, HERV-W, HERV-F, ERV-9, HERV-K (HML-2, -3, -4, -7, and -8 groups), and HERV-L groups, and in HUVEC cells from healthy donors, ERV-9, HERV-F, and HERV-K (HML-2, -5, and -6 groups) were upregulated under HCMV infection condition^68^. Furthermore, in kidney transplant recipients, HCMV induces HERV-K and HERV-W expression, demonstrating its clinical relevance^69^. However, lytic replication of HCMV may not be the reason of HERV-K expression due to no inhibitory effect of blocking HCMV replication by ganciclovir or silencing of IE1/IE2 on HERV-K transactivation^68^. Interestingly, in contrast to KSHV, UV-inactivated HCMV still activates HERV-K expression, but the increase in the HERV-K activity is far less pronounced than in normal HCMV infection^68^. Thus, activation of HERVs by HCMV infection may be directly induced through some cytokines and/or growth factors in response to viral infection.

### HTLV-1 infection and HERVs transactivation

Human T-lymphotropic virus 1 (HTLV-1) belongs to a group of human retroviruses and is known as the causative agent in adult T-cell leukemia (ATL) and HTLV-1-associated myelopathy/tropical spastic paraparesis (HAM/TSP)^70^. Although most HTLV-1 infected patients maybe asymptomatic throughout their lives, this virus is now estimated to infect 5–10 million people worldwide^71^. The viral Tax protein has been considered to play an important role in the development of HTLV-1-associated diseases. HTLV-1 Tax protein performs the powerful function of activator to modulate the expression of many viral and cellular genes, such as CREB, NF-κB, and SRF^72,73^.

It has been found that HTLV-1 Tax protein activates LTR of several HERVs, including HERV-W, HERV-H, HERV-E, and HERV-K families, speculating a potential link between HERVs transactivation and HTLV-1-associated diseases^35^. Moreover, the data from HLTV-1 infected patients show an increased prevalence of antibodies to Pol and Gag peptides of the retrovirus HERV-K10, homologous to HTLV-1 gp21 envelope and p24 Gap protein, respectively^74^. This prevalence was observed to be higher in HLTV-1 infected patients with myelopathy (87%) vs non-myelopathy (5.2%)^74^. Thus, HTLV-1 Tax-activated HERVs and/or HTLV-1-induced immuno-cross-reactivity may be involved in the pathogenesis of these virus-associated diseases.

### HBV infection and HERVs transactivation

Hepatitis B virus (HBV), a small double-stranded DNA virus, causes acute and chronic hepatitis B in humans^75^. Chronic hepatitis B caused by HBV infection is the major cause of HCC worldwide, and remains therefore a major public health problem globally^75,76^. HBV-encoded X protein (HBx) is believed to be a potent regulator in the pathogenesis of HBV-related HCC^77^.

HBx is a multifunctional oncogenic protein that modulates and activates the expression of many viral and cellular factors^77^. A recent study showed that HBx increased the promoter activity of HERV-W *env* to up-regulate its expression through the NF-κB pathway in human hepatoma HepG2 cells^34^. However, elucidating the function of HERVs transactivation in HBV-induced HCC still requires further investigation. Although the association of HERVs transactivation with HBV-induced HCC remains largely unclear, an interesting study showed that HERV-K transactivation is correlated with the prognosis and progress of HCC^17^. These data may provide a new insight about HERVs transactivation in HBV-associated HCC development.

## Conclusion

In contrast to other “conventional” cancers, the role of HERVs transactivation in viral oncogenesis remains largely unknown. In recent years, the mechanisms of tumor virus-induced HERVs transactivation have been partially explored: (1) Virus-mediated transcriptional factors—the LTR regions of HERVs carry binding sites for many transcriptional factors (e.g., NF-κB), which can be activated by viral products and result in the induction of HERVs gene expression^78,79^. For example, KSHV LANA, EBV LMP-1, and HBV HBx can induce HERV transactivation through the NF-κB signaling pathway^33,34,57^. HLTV-1 Tax is also a classical activator of gene expression through modulating NF-κB activity, contributing to Tax-induced HERVs transactivation^35^. (2) Viral products directly regulate HERVs transactivation—many viral products can bind to the promoters of viral or host genes to regulate gene expression as transcriptional factors, such as EBV EBNAs and KSHV LANA^80,81^. Therefore, these viral products may directly bind to the LTR regions of HERVs to mediate their transactivation, although these still need experimental evidence support. (3) Viral infection-induced epigenetic modification—DNA tumor viruses have developed various mechanisms to affect the status of chromosome modification through the modulation of some key enzymes activities, such as DNA methyltransferase and histone deacetylase, further regulating viral and host gene expression^82^. For example, KSHV vFLIP can induce *AXL* expression potentially through *AXL* gene hypomethylation^82,83^. However, DNA methylation is considered as an important mechanism for silencing of HERVs, and hypomethylation in tumors and/or treatment with DNA-demethylating agents, such as 5-aza-2-deoxycytidine and 5-azacytidine, may lead to HERVs transactivation^84,85^. Therefore, a change in the epigenetic modification induced by viral infection may drive HERV transactivation. (4) Modification of host immune system by viral infection—previous studies have shown that the antibodies of HERVs were found in the sera of patients with autoimmune diseases, such as multiple sclerosis, rheumatoid arthritis, and lupus erythematosus, indicating the association between HERVs transactivation and host immune system^6,12^. Interestingly, the envelop protein of HERVs, such as HERV-H and HERV-K family, displays immunosuppressive properties in vivo^86,87^. DNA tumor viruses have developed various mechanisms to regulate host immune system^82,88^. Thus, virus-mediated host immune system modification may cause HERV transactivation, which in turn, contributes to the development of virus-associated malignancies.

Increasingly, recent literature supports that HERVs transactivation may be a potential contributor to the development of virus-associated tumors. Thus, studies on HERVs transactivation by different tumor viruses might provide new insights and strategies for the prevention and/or treatment of these special malignancies.
